# Xanthine oxidase inhibitors are associated with reduced risk of cardiovascular disease

**DOI:** 10.1038/s41598-020-80835-8

**Published:** 2021-01-14

**Authors:** Hirotaka Saito, Kenichi Tanaka, Tsuyoshi Iwasaki, Akira Oda, Shuhei Watanabe, Makoto Kanno, Hiroshi Kimura, Michio Shimabukuro, Koichi Asahi, Tsuyoshi Watanabe, Junichiro James Kazama

**Affiliations:** 1 Department of Nephrology and Hypertension, Fukushima Medical University, 1, Hikarigaoka, Fukushima, Japan; 2 Department of Chronic Kidney Disease Initiatives, Fukushima Medical University, Fukushima, Japan; 3 Department of Diabetes, Endocrinology and Metabolism, Fukushima Medical University, Fukushima, Japan; 4 Division of Nephrology and Hypertension, Iwate Medical University, Yahaba, Japan

**Keywords:** Outcomes research, Cardiovascular diseases, Risk factors, Metabolic disorders

## Abstract

As previous studies have reported finding an association between hyperuricemia and the development of cardiovascular and chronic kidney disease, hyperuricemia is thought to be an independent risk factor for hypertension and diabetic mellitus. However, we have not been able to determine whether the use of xanthine oxidase inhibitors can reduce cardiovascular disease. The present study used the longitudinal data of the Fukushima Cohort Study to investigate the relationship between the use of xanthine oxidase inhibitors and cardiovascular events in patients with cardiovascular risks. During the 3-year period between 2012 and 2014, a total of 2724 subjects were enrolled in the study and followed. A total of 2501 subjects had hypertension, diabetic mellitus, dyslipidemia, or chronic kidney disease, and were identified as having cardiovascular risks. The effects of xanthine oxidase inhibitor use on the development of cardiovascular events was evaluated in these patients using a time to event analysis. During the observational periods (median 2.7 years), the incidence of cardiovascular events was 20.7 in subjects with xanthine oxidase inhibitor and 11.2 (/1000 person-years, respectively) in those without. Although a univariate Cox regression analysis showed that the risk of cardiovascular events was significantly higher in subjects administered xanthine oxidase inhibitors (HR = 1.87, 95% CI 1.19–2.94, p = 0.007), the risk was significantly lower in subjects administered a xanthine oxidase inhibitor after adjustment for covariates (HR = 0.48, 95% CI 0.26–0.91; p = 0.024) compared to those without. Xanthine oxidase inhibitor use was associated with reduced risk of cardiovascular disease in patients with cardiovascular risk factors.

## Introduction

Recent epidemiological studies have demonstrated an association between serum uric acid levels and hypertension, diabetic mellitus, or metabolic syndrome. Moreover, results of meta-analyses have also shown that hyperuricemia itself exhibited a relationship with the new onset of hypertension and diabetes, and thus, has been proposed as being an independent risk factor of these diseases^1–5^. Hypertension, diabetes, and metabolic syndrome are well-recognized as risk factors for atherosclerosis and cardiovascular disease. In addition, hyperuricemia has been reported to have an independent relationship with the risk for progression of cardiovascular events and chronic kidney disease (CKD)^6–14^. Goicoechea et al.^15^ reported that a lowering of the serum uric acid by allopurinol was able to slow down the progression of renal disease and reduced the cardiovascular risk in a randomized controlled trial with a limited number of patients with CKD. Terawaki et al.^16^ additionally showed in an observational cohort study (Gonryo cohort) that the use of xanthine oxidase inhibitor (XOI) was associated with a lower risk of cardiovascular morbidity in hypertensive subjects with impaired kidney function. However, as this previous study only examined a limited number of subjects in this specific patient group, it remains unclear as to whether the use of XOI for treating hyperuricemia can reduce the risk of cardiovascular disease in the high-risk group with relevant risk factors, such as hypertension, diabetes, dyslipidemia, and CKD. The objective of the present study was to evaluate the relationship between the use of XOIs and a reduced risk of cardiovascular events in a cohort of high-risk patients with hypertension, diabetes, dyslipidemia, and CKD (Fukushima Cohort Study).

## Material and methods

### Study population (Fukushima Cohort)

The Fukushima Cohort Study is a prospective survey of patient characteristics and outcomes for subjects who are being followed at the Fukushima Medical University Hospital (Fukushima Prefecture, northeastern area of Japan). Patient enrollment was conducted between June 2012 and July 2014, with a total of 2724 patients registered for the study.

Inclusion criteria were as follows: (1) Japanese patient living in Japan, (2) aged 18 years or older, and (3) patients having one or more cardiovascular risk factors, such as CKD, hypertension, diabetes, and dyslipidemia.

Exclusion criteria were as follows: (1) receiving renal replacement therapy in the last three months, (2) active malignancy, (3) infectious disease, (4) pregnancy, (5) type 1 diabetes mellitus, and (6) history of organ transplantation.

**Figure 1:**
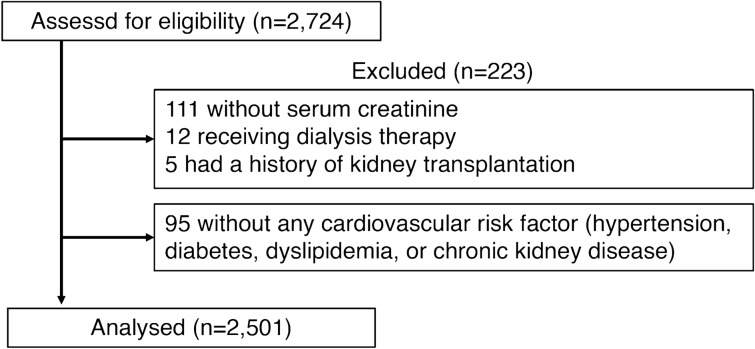
A flowchart representing the participants’ inclusion into the present study.

A total of 2501 cases were identified as patients with cardiovascular risk and analyzed (Fig. 1). The protocol was approved by the Ethics Committee of Fukushima Medical University, and the study was conducted in accordance with the Declaration of Helsinki. All patients provided written informed consent.

### Data collection and primary outcomes

Serum creatinine was measured by an enzyme assay method. Estimated GFR (eGFR) was calculated using an estimation formula specifically designed for Japanese subjects^17^. Serum uric acid, serum albumin, LDL cholesterol, and HDL cholesterol levels were measured according to the automated standardized laboratory technique of the clinical laboratory of our institution. Urinary protein levels were calculated using spot urine specimens and adjusted for creatinine (g/gCr).

Blood pressure was measured using a standard sphygmomanometer with the patient in a sitting position. Body mass index was calculated as weight (kg) divided by height squared (m^2^). CKD was defined as an eGFR < 60 mL/min/1.73 m^2^ or proteinuria ≥ (1 +) by dipstick, with a stable renal function present for at least three months before enrollment. Hypertension was defined as follows: (1) systolic blood pressure ≥ 140 mmHg, or (2) diastolic blood pressure ≥ 90 mmHg, or the use of antihypertensive medication. Subjects with diabetes mellitus were classified as follows: (1) fasting plasma glucose concentration ≥ 126 mg/dL, (2) hemoglobin A1c value (National Glycohemoglobin Standardization Program) ≥ 6.5%, or (3) patients who use insulin or oral antihyperglycemic drugs, and have dyslipidemia that presented with values as follows: (1) triglyceride ≥ 150 mg/dL, (2) LDL cholesterol concentration ≥ 140 mg/dL, (3) HDL cholesterol concentration < 40 mg/dL, or (4) use of antihyperlipidemic medication. A questionnaire survey was conducted on smoking status at baseline, and the status was classified as never smoker, past smoker, or current smoker.

Information regarding medication at baseline, or past medical histories such as cardiovascular disease, hypertension, diabetes mellitus, and hyperuricemia were derived from the medical records or from the results of blood examinations performed at the time of enrollment in the study.

Primary outcome of this study was the development of cardiovascular events, including fatal or nonfatal myocardial infarction, angina pectoris, sudden death, congestive or acute heart failure, arrhythmias, cerebrovascular disorder, chronic arteriosclerosis obliterans, and aortic disease.

### Statistical analyses

Categorical variables are expressed as percentages while the continuous variables are presented as the median and interquartile range. SPSS software (version 26; IBM Corporation, Chicago, IL, USA) was used for all statistical analysis. Values of P < 0.05 were considered statistically significant. Values were compared using a Mann–Whitney’s U test or χ^2^ test, where appropriate. The association between the use of XOI and cardiovascular events was examined using a Cox proportional hazard model analysis, with adjustments made for confounding factors.

## Results

Table 1 presents the baseline characteristics of subjects. XOIs were used in 19.0% of all subjects, for which 44.4% were administered allopurinol and 55.6% febuxostat. In XOI (+) group, 81.3% of subjects were administered angiotensin converting enzyme inhibitor (ACEi) or angiotensin II receptor blocker (ARB), of whom 32.1% had cardiovascular disease, while 44.9% of XOI (+) without ACEi or ARB had cardiovascular disease.

**Table 1:** Characteristics of the study patients at baseline.

Variables	All patients	XOI		p
		−	+	
N	2501	2026	475	
Age (years)	64 [54–72]	63 [53–72]	64 [55–74]	0.015
Male sex (%)	51.1	45.6	74.3	< 0.001
Body mass index (kg/m^2^)	24.3 [21.8–27.2]	24.0 [21.6–27.0]	25.3 [22.9–27.9]	< 0.001
BMI ≥ 25 kg/m^2^ (%)	43.0	40.3	54.0	< 0.001
**Smoking status (%)**				
Never/past/current	48.6/32.1/14.6	51.9/30.7/13.5	34.5/37.9/19.2	< 0.001
Cardiovascular disease (%)	23.6	21.1	34.5	< 0.001
Systolic blood pressure (mmHg)	131 [120–143]	131 [120–143]	131 [120–143]	0.899
Diastolic blood pressure (mmHg)	76 [68–84]	76 [68–84]	77 [69–84]	0.127
Serum creatinine (mg/dL)	0.81 [0.65–1.05]	0.76 [0.63–0.93]	1.26 [0.98–1.76]	< 0.001
Estimated GFR (mL/min./1.73 m^2^)	65.3 [50.5–79.8]	69.4 [57.5–82.4]	42.1 [27.6–57.3]	< 0.001
Proteinuria (g/gCr)	0.79 [0.00–0.47]	0.04 [0.00–0.26]	0.30 [0.02–1.18]	< 0.001
Hypertension (%)	15.7	15.9	14.7	0.516
Dyslipidemia (%)	75.1	73.2	83.0	< 0.001
Diabetes (%)	49.3	50.5	44.4	0.017
Serum albumin (g/dL)	4.0 [3.8–4.3]	4.1 [3.8–4.3]	3.9 [3.6–4.2]	< 0.001
Hemoglobin (g/dL)	13.2 [12.1–14.3]	13.2 [12.2–14.2]	13.1 [11.4–14.4]	0.003
Uric acid (mg/dL)	5.6 [4.6–6.6]	5.4 [4.5–6.4]	6.4 [5.4–7.4]	< 0.001
LDL-cholesterol (mg/dL)	105 [87–125]	106 [88–126]	101 [80–120]	< 0.001
HDL-cholesterol (mg/dL)	53 [44–63]	54 [45–65]	47 [39–55]	< 0.001
Type of XOI allopurinol/febuxostat (%)	NA	NA	44.4/55.6	
ACEi or ARB (%)	58.3	52.9	81.3	< 0.001
Diuretics (%)	20.6	16.8	36.6	< 0.001
Aspirin (%)	12.8	11.4	18.7	< 0.001
Warfarin (%)	5.2	3.9	10.5	< 0.001
Statin (%)	44.0	43.0	48.2	0.041
Calcium channel blocker (%)	47.6	44.0	63.2	< 0.001
β blocker	12.1	9.5	23.2	< 0.001

Categorical variables are expressed as percentages and continuous variables as median [interquartile range].

*XOI* xanthine oxidase inhibitor, *BMI* body mass index, *GFR* glomerular filtration rate, *LDL* low density lipoprotein, *HDL* high density lipoprotein, *ARB* angiotensin II receptor blocker, *ACEi* angiotensin converting enzyme inhibitor.

During the median observational periods of 2.7 [interquartile range 2.2–3.7] years, 86 out of 2501 patients developed cardiovascular events, for which 28 patients were being administered XOI. Table 2 shows the details for these events.

**Table 2:** Details of cardiovascular events.

	Number of patients		
	All	XOI	
		−	+
Congestive and acute heart failure	29	17	12
Cerebral infarction	18	11	7
Myocardial infarction	12	9	3
Angina pectoris	9	5	4
Aortic disease	6	4	2
Cerebral hemorrhage	4	4	0
Subarachnoid hemorrhage	3	3	0
Arrhythmia	2	2	0
Chronic arteriosclerosis obliterans	2	2	0
Sudden death	1	1	0
Total	86	58	28

*XOI* xanthine oxidase inhibitor.

The incidence of cardiovascular events was higher in the XOI (+) versus the XOI (−) group (20.7 vs. 11.2/1,000 person-years). Particularly in XOI (+) group, the incidence was lower in patients with ACEi or ARB than those without (18.9 vs. 28.7/1000 person-years). The univariate Cox regression analysis showed that there was a significantly higher risk in the XOI (+) versus the XOI (−) group (HR = 1.87, 95% confidence interval (CI) 1.19–2.94, p = 0.007). For the multivariate analysis, we adapted the following covariates; age, sex, and eGFR (Model 1), age, sex, eGFR, history of cardiovascular disease, diabetes, smoking status, systolic blood pressure, and serum uric acid (Model 2), and age, sex, eGFR, history of cardiovascular disease, diabetes, smoking status, systolic blood pressure, serum uric acid, BMI ≧ 25, proteinuria, and the use of diuretics (Model 3). Table 3 presents the results. The adjusted hazard ratio for the use of XOI when cardiovascular events occurred was 0.63 (95% CI 0.38–1.06, p = 0.081) in Model 1, 0.56 (95% CI 0.33–0.97; p = 0.039) in Model 2, and 0.48 (95% CI 0.26–0.91; p = 0.023) in Model 3. The use of XOI had a still significant effect on cardiovascular disease after adjustment by covariates in Model 3 plus use of ACEi or ARB (Supplemental Table 1).

**Table 3:** Cox regression analyses of independent factors for cardiovascular events.

Variables	Univariate			Model 1			Model 2			Model 3		
	HR	95% CI	p	HR	95% CI	p	HR	95% CI	p	HR	95% CI	p
XOI	1.87	1.19–2.94	0.007	0.63	0.38–1.06	0.081	0.56	0.33–0.97	0.039	0.48	0.26–0.91	0.023
Age	1.06	1.04–1.08	< 0.001	1.04	1.02–1.06	< 0.001	1.01	0.98–1.03	0.692	0.98	0.96–1.01	0.280
Sex	2.08	1.32–3.26	0.002	1.91	1.21–2.99	0.005	1.77	0.92–3.42	0.087	1.85	0.86–4.00	0.115
eGFR (mL/min./1.73 m^2^)	0.96	0.95–0.97	< 0.001	0.97	0.96–0.98	< 0.001	0.96	0.94–0.97	< 0.001	0.96	0.94–0.97	< 0.001
Cardiovascular disease	8.50	5.35–13.48	< 0.001	5.38	3.28–8.83	< 0.001	6.74	3.77–12.1	< 0.001	9.38	4.46–19.7	< 0.001
Diabetes	2.20	1.40–3.44	0.001	2.08	1.33–3.27	0.001	1.56	0.93–2.63	0.094	2.05	1.03–3.94	0.032
**Smoking status**												
Never	ref			ref			ref			ref		
Past	2.48	1.53–4.02	< 0.001	1.59	0.87–2.90	0.133	1.30	0.70–2.43	0.402	1.45	0.71–2.98	0.312
Current	1.19	0.56–2.53	0.655	1.30	0.57–2.97	0.533	1.31	0.56–3.04	0.530	1.74	0.70–4.31	0.235
Systolic blood pressure (mmHg)	1.01	1.00–1.02	0.246	1.01	1.00–1.02	0.181	1.01	1.00–1.02	0.086	1.01	1.00–1.03	0.084
Serum uric acid (mg/dL)	1.25	1.08–1.44	0.003	0.96	0.81–1.13	0.598	0.93	0.78–1.11	0.430	0.84	0.68–1.03	0.096
BMI ≥ 25 kg/m^2^	1.45	0.93–2.26	0.102	1.20	0.77–1.88	0.423				1.61	0.88–2.95	0.120
Proteinuria (g/gCr)	1.10	0.97–1.25	0.141	0.99	0.83–1.18	0.890				0.94	0.76–1.15	0.518
Diuretics	3.06	2.00–4.68	< 0.001	1.87	1.20–2.90	0.005				1.72	0.97–3.07	0.064

Model 1; adjusted for age, sex, and eGFR. Model 2; adjusted for age, sex, eGFR, history of cardiovascular disease, diabetes, smoking status, systolic blood pressure, and serum uric acid. Model 3; adjusted for age, sex, eGFR, history of cardiovascular disease, diabetes, smoking status, systolic blood pressure, serum uric acid, BMI ≥ 25 kg/m^2^, proteinuria (g/gCr), and use of diuretics.

*XOI* xanthine oxidase inhibitor, *eGFR* estimated glomerular filtration rate, *BMI* body mass index, *HR* hazard ratio, *CI* confidence interval.

**Figure 2:**
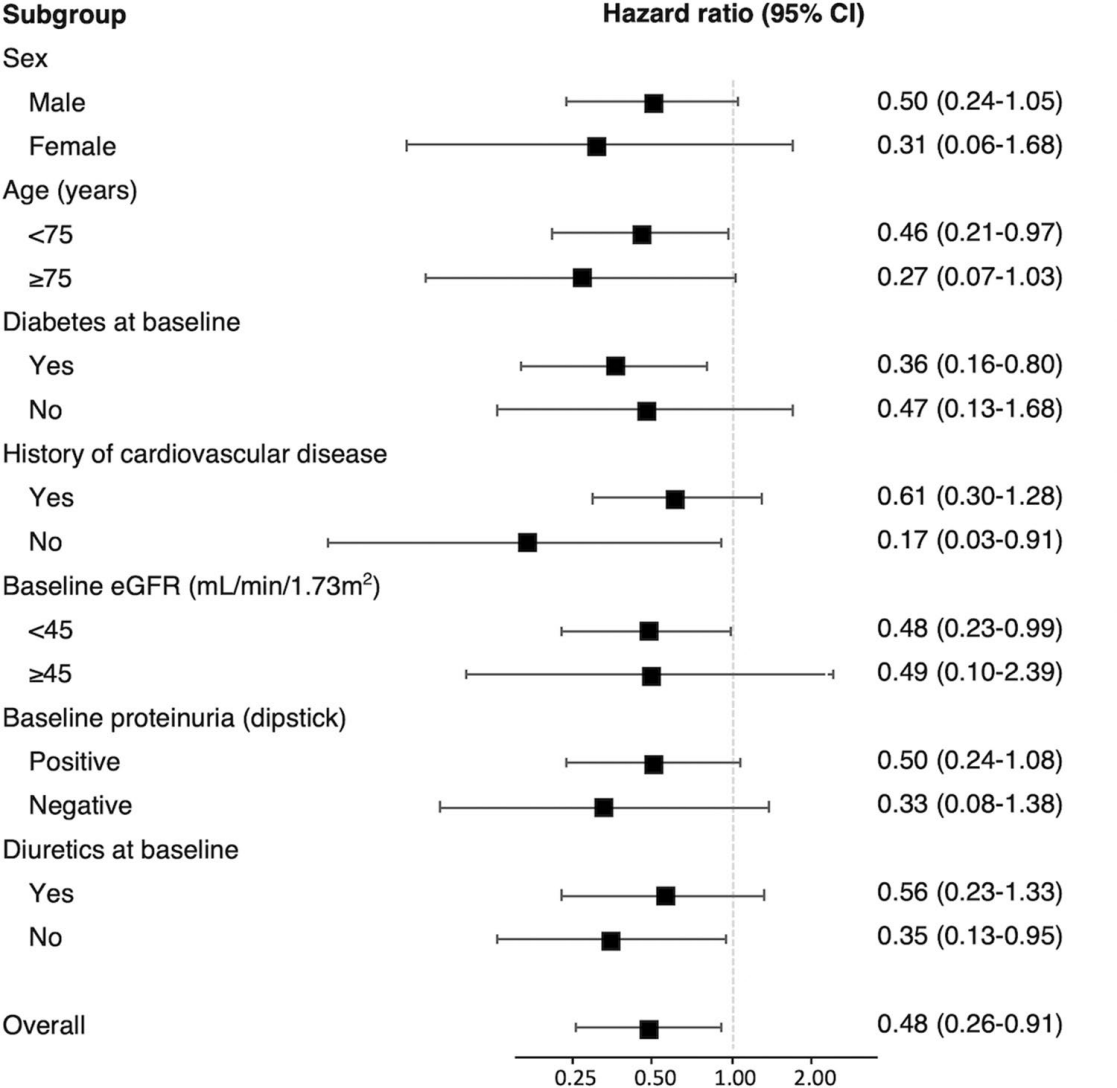
Hazard ratios of XOI use for the cardiovascular events for the subgroup according to the baseline variates. Hazard ratios were adjusted for age, sex, eGFR, history of cardiovascular disease or diabetes, smoking status, systolic blood pressure, serum uric acid, BMI ≥ 25 kg/m^2^, proteinuria (g/gCr), and use of diuretics. *XOI* xanthine oxidase inhibitor, *CI* confidence interval, *eGFR* estimated glomerular filtration rate, *BMI* body mass index.

Figure 2 shows the hazard ratios of XOI use for the cardiovascular events for the subgroup according to the baseline variates (covariates are the same as those shown for Model 3 in Table 2). The risk for cardiovascular disease was significantly lower in age group evaluated < 75 years, those with diabetes, without history of cardiovascular disease, eGFR < 45 ml/min/1.73 m^2^, and diuretics non-user groups.

## Discussion

For the first time, this large observational cohort study showed there was an association between the use of XOI and a lower risk of developing cardiovascular events in patients with existing cardiovascular risks such as hypertension, diabetic mellitus, dyslipidemia, or CKD. Although the incidence of new onset of cardiovascular events was significantly higher in the patients receiving XOI therapy, the use of XOI was significantly associated with a lower risk of cardiovascular morbidity that was independent of serum urate levels and other covariates after multivariate adjustment. This suggests that XOI may have a protective effect with regard to the development of cardiovascular disease.

In XOI (+) group, 81.3% of subjects were treated with ACEi or ARB, and the incidence rate of cardiovascular events was lower in subjects with ACEi or ARB than those without. As both ACEi and ARB reportedly reduced the cardiovascular risk^18, 19^, the lower risk of cardiovascular events in the present study might be related to the effects of these drugs. Therefore, multivariate adjustment was conducted in the model including use of ACEi or ARB as a covariate, and the result suggested that the use of XOI had an independent effect on lower risk of cardiovascular disease in these patients.

Subgroup analysis was also conducted and revealed that the lower risk of cardiovascular events was relevant in patients with diabetes, or in those patients with a decreased eGFR, which indicates that the protective effect of XOI for cardiovascular disease would be expected to occur in patients with a higher risk of cardiovascular disease. Furthermore, as compared to patients with cardiovascular disease history, XOI use in those without any history was significantly associated with a lower risk of cardiovascular disease. Further investigations will need to be undertaken in order to clarify the effect of XOI as secondary prevention of cardiovascular disease. However, the lower rate of the patients with cardiovascular disease history that were present at baseline in the current study could have potentially affected our results.

Recently, two large randomized controlled trials were conducted. The Febuxostat for Cerebral and CaRdiorenovascular Events PrEvEntion StuDy (FREED) study was a randomized controlled trial that compared the effect of febuxostat with conventional therapy with regard to the risks of the primary composite endpoints, which included cerebral, cardiovascular, and renal events in patients with hyperuricemia^20^. Although the primary composite event rate was significantly lower in the febuxostat group, there were no beneficial effects found for the cardiovascular and cerebrovascular events during a subgroup analysis. However, since this study was not a placebo-controlled trial, these results do not necessarily indicate that XOI did not reduce the risk of cardiovascular disease.

In the second trial, the Cardiovascular Safety of Febuxostat or Allopurinol in Patients with Gout (CARES) study found that the all-cause and cardiovascular mortalities were higher for febuxostat as compared to allopurinol in patients with gout and major cardiovascular coexisting conditions^21^. Even so, these results do not necessarily demonstrate that febuxostat was harmful, as the risk of cardiovascular disease was compared to allopurinol and not to the placebo. In fact, the cardiovascular risk was significantly lower in the present study in patients administered XOIs versus those that did not, even though this study had a larger administration of febuxostat (55.6%). However, the effects of XOIs on cardiovascular risk reduction will need to be investigated for allopurinol, febuxostat, and topiroxostat separately in well-designed future study.

Our current study also showed that there was a significant association between XOI use and a lower cardiovascular risk in patients not using diuretics as compared to those who were. This finding could potentially indicate that the secondary rising of serum urate levels due to the use of diuretics does not necessarily affect the development of cardiovascular morbidity. Hozawa et al.^22^ reported that serum uric acid levels were related to ischemic stroke incidence in the Atherosclerosis Risk in Communities (ARIC) Study. Although this relationship was only found to be significant among subjects who were not using diuretics, the authors hypothesized that diuretic-induced hyperuricemia did not cause ischemic stroke, which is unlike hyperuricemia due to oxidative stress that can potentially be harmful.

It has been previously suggested that the protective effect mechanism of XOI for cardiovascular disease might be associated with a reduction of oxidative stress generated by xanthine oxidase during the metabolism of uric acid, thereby protecting endothelial cells from the progression of atherosclerosis^23–25^. The protective effects of XOI on blood vessels or cardiovascular system are also demonstrated in some studies of animal models^26–28^. The present study shows higher serum uric acid level in XOI (+) group than in XOI (−) group at baseline, and it might support above direct effect mechanism of XOI by inhibiting XO activity. However, it remains unknown as to whether the reduction of oxidative stress by XOI is derived from a lowering of the serum urate level or due to an effect related to the inhibition of xanthine oxidase itself. Therefore, more experiments including both basic and clinical research will need to be undertaken in order to definitively elucidate this mechanism.

There were several limitations for the current study. First, since this was an observational cohort study, the effect on cardiovascular risk reduction by XOIs will need to be confirmed in well-designed interventional study. Second, while allopurinol and febuxostat were used for XOIs in this study, the novel selective xanthine oxidoreductase inhibitor, topiroxostat, was not examined. Furthermore, we did not consider the doses of allopurinol or febuxostat used in the present study. Although it has been previously reported that the dose of allopurinol needs to be restricted in patients with renal dysfunction in order to avoid the severe side effects, the beneficial effects on the endothelial function depend on the actual dose^29^. In addition, as the subjects of the present cohort contained many patients with chronic kidney disease, febuxostat or topiroxostat might be more ideal for investigating the effect of XOIs rather than allopurinol. Third, detailed information on obesity and smoking are limited in the present study, in spite of the fact that both obesity and smoking are known to be classical risk factors for cardiovascular disease. Waist-to-height ratio, waist-to-hip ratio, and waist circumference were reportedly better predictive values for cardiovascular events and death than BMI^30–32^. Smoking intensity, such as pack/day, was reportedly a better measure for cardiovascular risk than smoking status (never/past/current). These detailed data for assessing obesity and smoking were not available in the present study. Finally, as this study only confirmed the levels of serum uric acid at baseline, it is unknown whether the lowering of the serum urate levels after treatment could have affected the results for the reduced risk of cardiovascular events.

Kimura et al.^33^ demonstrated that febuxostat did not exhibit any renoprotective effect on the decline of eGFR in any of the subjects as compared to the placebo in the Febuxostat Therapy for Patients With Stage 3 CKD and Asymptomatic Hyperuricemia: A Randomized Trial (FEATHER study). However, the subgroup analysis showed that febuxostat had a significant benefit in patients without proteinuria and for whom the serum creatinine was lower than the median. Furthermore, Terawaki et al.^34^ reported finding renoprotective effects of topiroxostat via the reduction of albuminuria after switching from febuxostat. However, in all of the above studies, the reports of protective effects of XOIs in preventing progression of CKD have only been demonstrated in a limited number of patients. Thus, although only a few studies have been able to confirm the protective effect of XOIs with regard to the cardiovascular system in random controlled trials, XOIs might be able to reduce the risk of developing cardiovascular events by preventing CKD, which is one of the major risk factors of cardiovascular disease.

In conclusion, the use of XOI in pre-dialysis patients with cardiovascular risks may provide a beneficial effect of reducing cardiovascular events. In the future, we will need to conduct a randomized controlled trial that investigates the effect of XOIs that contain febuxostat or topiroxostat for not only the risks of cardiovascular events, but also with regard to mortality or progression of end stage renal disease in these types of patients.

## Supplementary Information

Supplementary Table 1.

## References

[1] Grayson PC, Kim SY, LaValley M, Choi HK (2011). Hyperuricemia and incident hypertension: A systematic review and meta-analysis. Arthritis Care Res. (Hoboken).

[2] Wang J (2014). Hyperuricemia and risk of incident hypertension: A systematic review and meta-analysis of observational studies. PLoS ONE.

[3] Sui X, Church TS, Meriwether RA, Lobelo F, Blair SN (2008). Uric acid and the development of metabolic syndrome in women and men. Metabolism.

[4] Onat A (2006). Serum uric acid is a determinant of metabolic syndrome in a population-based study. Am. J. Hypertens..

[5] Yu TY (2016). Serum uric acid: A strong and independent predictor of metabolic syndrome after adjusting for body composition. Metabolism.

[6] Obermayr RP (2008). Elevated uric acid increases the risk for kidney disease. J. Am. Soc. Nephrol..

[7] Iseki K (2004). Significance of hyperuricemia as a risk factor for developing ESRD in a screened cohort. Am. J. Kidney Dis..

[8] Kamei K (2014). A slight increase within the normal range of serum uric acid and the decline in renal function: Associations in a community-based population. Nephrol. Dial. Transpl..

[9] Tanaka K (2015). Renoprotective effects of febuxostat in hyperuricemic patients with chronic kidney disease: A parallel-group, randomized, controlled trial. Clin. Exp. Nephrol..

[10] Braga F, Pasqualetti S, Ferraro S, Panteghini M (2016). Hyperuricemia as risk factor for coronary heart disease incidence and mortality in the general population: A systematic review and meta-analysis. Clin. Chem. Lab. Med..

[11] Li M (2016). Hyperuricemia and the risk for coronary heart disease morbidity and mortality a systematic review and dose-response meta-analysis. Sci. Rep..

[12] Huang H (2014). Uric acid and risk of heart failure: A systematic review and meta-analysis. Eur. J. Heart Fail..

[13] Uchida S (2015). Targeting uric acid and the inhibition of progression to end-stage renal disease—A propensity score analysis. PLoS ONE.

[14] Zhang CH (2016). Association between serum uric acid levels and atrial fibrillation risk. Cell Physiol. Biochem..

[15] Goicoechea M (2015). Allopurinol and progression of CKD and cardiovascular events: Long-term follow-up of a randomized clinical trial. Am. J. Kidney Dis..

[16] Terawaki H (2013). Effect of allopurinol on cardiovascular incidence among hypertensive nephropathy patients: The Gonryo study. Clin. Exp. Nephrol..

[17] Matsuo S (2009). Revised equations for estimated GFR from serum creatinine in Japan. Am. J. Kidney Dis..

[18] Yusuf S, Heart Outcomes Prevention Evaluation Study Investigators (2000). Effects of an angiotensin-converting–enzyme inhibitor, ramipril, on cardiovascular events in high-risk patients. N. Engl. J. Med..

[19] Liebson PR, Amsterdam EA (2009). Ongoing telmisartan alone and in combination with ramipril global endpoint trial (ONTARGET): Implications for reduced cardiovascular risk. Prevent. Cardiol..

[20] Kojima S (2019). Febuxostat for cerebral and CaRdiorenovascular Events PrEvEntion StuDy. Eur. Heart J..

[21] White WB (2018). Cardiovascular safety of febuxostat or allopurinol in patients with gout. N. Engl. J. Med..

[22] Hozawa A (2006). Serum uric acid and risk of ischemic stroke: the ARIC Study. Atherosclerosis.

[23] Clarson LE (2015). Increased cardiovascular mortality associated with gout: A systematic review and meta-analysis. Eur. J. Prev. Cardiol..

[24] Heinig M, Johnson RJ (2006). Role of uric acid in hypertension, renal disease, and metabolic syndrome. Cleve Clin. J. Med..

[25] Zoppini G, Targher G, Bonora E (2011). The role of serum uric acid in cardiovascular disease in type 2 diabetic and non-diabetic subjects: A narrative review. J. Endocrinol. Invest..

[26] Mazzali M (2001). Elevated uric acid increases blood pressure in the rat by a novel crystal-independent mechanism. Hypertension.

[27] Sanchez-Lozada LG (2008). Effects of febuxostat on metabolic and renal alterations in rats with fructose-induced metabolic syndrome. Am. J. Physiol. Renal. Physiol..

[28] Zhao L (2008). Chronic xanthine oxidase inhibition following myocardial infarction in rabbits: Effects of early versus delayed treatment. Life Sci..

[29] George J, Carr E, Davies J, Belch JJ, Struthers A (2006). High-dose allopurinol improves endothelial function by profoundly reducing vascular oxidative stress and not by lowering uric acid. Circulation.

[30] Nance R (2017). Smoking intensity (pack/day) is a better measure than pack-years or smoking status for modeling cardiovascular disease outcomes. J. Clin. Epidemiol..

[31] DeVallance E (2015). Is obesity predictive of cardiovascular dysfunction independent of cardiovascular risk factors?. Int. J. Obes. (Lond.).

[32] Schneider HJ (2010). The predictive value of different measures of obesity for incident cardiovascular events and mortality. J. Clin. Endocrinol. Metab..

[33] Kimura K (2018). Febuxostat therapy for patients with stage 3 CKD and asymptomatic hyperuricemia: A randomized trial. Am. J. Kidney Dis..

[34] Terawaki H, Hoshi H, Kazama JJ (2017). Effect of switching xanthine oxidoreductase inhibitor from febuxostat to topiroxostat on urinary protein excretion. Clin. Exp. Nephrol..

