# Supplementary material for: Xanthine oxidase inhibitors are associated with reduced risk of cardiovascular disease
Source: Sci Rep. 2021 Jan 14;11:1380. doi: 10.1038/s41598-020-80835-8 (PMC7809289; doi:10.1038/s41598-020-80835-8)
Supplement: Supplementary file 1 — Supplementary Table 1. [file 41598_2020_80835_MOESM1_ESM.docx]

Title page

**Xanthine oxidase inhibitors are associated with reduced risk of cardiovascular disease**

Hirotaka Saito^1^, *Kenichi Tanaka^1^, Tsuyoshi Iwasaki^1^, Akira Oda^1^, Shuhei Watanabe^1^, Makoto Kanno^2^, Hiroshi Kimura^1^, Michio Shimabukuro^3^, Koichi Asahi^4^, Tsuyoshi Watanabe^2^, Junichiro James Kazama^1^

^1)^Department of Nephrology and Hypertension, Fukushima Medical University, Fukushima, ^2)^Department of Chronic Kidney Disease Initiatives, Fukushima Medical University, Fukushima, ^3)^Department of Diabetes, Endocrinology and Metabolism, Fukushima Medical University, Fukushima, and ^4)^Department of Nephrology and Hypertension, Iwate Medical University, Morioka, Japan.

Supplemental Table 1. Cox regression analyses of independent factors for cardiovascular events.

| Variables | Model 4 | | |
| --- | --- | --- | --- |
|  | HR | 95%CI | p |
| XOI | 0.49 | 0.26-0.93 | 0.029 |
| Age | 0.98 | 0.96-1.01 | 0.289 |
| Sex | 1.88 | 0.87-4.09 | 0.110 |
| eGFR (mL/min./1.73m^2^) | 0.96 | 0.94-0.97 | < 0.001 |
| Cardiovascular disease | 9.31 | 4.43-19.6 | < 0.001 |
| Diabetes | 2.03 | 1.05-3.90 | 0.034 |
| Smoking status |  |  |  |
| Never | ref |  |  |
| Current | 1.78 | 0.71-4.44 | 0.217 |
| Past | 1.44 | 0.70-2.98 | 0.321 |
| Systolic blood pressure (mmHg) | 1.01 | 1.00-1.03 | 0.075 |
| Serum uric acid (mg/dL) | 0.83 | 0.68-1.03 | 0.086 |
| BMI ≥ 25 kg/m^2^ | 1.55 | 0.83-2.89 | 0.167 |
| Proteinuria (g/gCr) | 0.94 | 0.77-1.15 | 0.534 |
| Diuretics | 1.77 | 0.99-3.17 | 0.056 |
| ACEi or ARB | 0.84 | 0.45-1.57 | 0.578 |

*XOI*; xanthine oxidase inhibitor, *eGFR*; estimated glomerular filtration rate, *ACEi*; angiotensin converting enzyme inhibitor, *ARB*; angiotensinⅡreceptor blocker, *HR*; hazard ratio, CI; confidence interval.

Model 4; adjusted for age, sex, eGFR, history of cardiovascular disease, diabetes, smoking status, systolic blood pressure, serum uric acid, proteinuria (g/gCr), use of diuretics and ACEi/ARB.
